# Otto Neurath’s Scientific Utopianism Revisited-A Refined Model for Utopias in Thought Experiments

**DOI:** 10.1007/s10838-022-09630-5

**Published:** 2023-03-23

**Authors:** Alexander Linsbichler, Ivan Ferreira da Cunha

**Affiliations:** 1 Department of Economics and Department of Philosophy, University of Vienna, Vienna, Austria; 2 Department of Philosophy, Federal University of Santa Catarina, Florianópolis, SC Brazil

**Keywords:** Otto Neurath, Utopia, Thought experiment, Argument view, Platonism, Policy advice

## Abstract

Otto Neurath’s empiricist methodology of economics and his contributions to political economy have gained increasing attention in recent years. We connect this research with contemporary debates regarding the epistemological status of thought experiments by reconstructing Neurath’s utopias as linchpins of thought experiments. In our three reconstructed examples of different uses of utopias/dystopias in thought experiments we employ a reformulation of Häggqvist’s model for thought experiments and we argue that: (1) Our reformulation of Häggqvist’s model more adequately complies with many uses of thought experiments, especially with the open-ended discussions of utopias and dystopias in thought experiments. (2) As a strict logical empiricist, Neurath is committed to a strictly empiricist account of thought experiments. John Norton’s empiricist argument view can indeed account for the justifications of empirical beliefs and genuine discoveries targeted by scientific utopianism in three distinct (yet connected) ways, all of which Neurath already contemplated: (2.I) Dealing with utopias and thought experiments on a regular basis increases creativity and inventiveness. (2.II) Particular ways of presenting knowledge facilitate scientific discovery and social progress. (2.III) The use of utopias in thought experiments can prompt conceptual change and allow access to new phenomena. We conclude by highlighting that, even though thought experiments support a positive attitude for exploring new social possibilities, Neurath points out that active decisions are unavoidable. The exploration of alternatives and the awareness of a need for decisions in policy discussion avert a technocratic outlook in social science.

## Introduction

Can we learn about the world just by exercising our imagination? One might answer negatively straight away. After all, our imagination can (at best) only provide knowledge of our own mental states. However, the use of thought experiments in investigations of domains as diverse as subatomic entities, consumer behavior, viral spread, personal identity, and moral responsibility seems to indicate otherwise. In the lively debate on “how can we learn about reality […] just by thinking?” (Brown and Fehige 2017), two seemingly extreme positions regarding thought experiments are in evidence: James Robert Brown’s Platonism, according to which some thought experiments provide intuition of universals, and John Norton’s empiricist stance sustaining that thought experiments are simply picturesque forms of arguments or, at least, that they are rationally reconstructible as such.^1^ The debate developed as attempts to find a middle ground between the two extremes were devised. A perspective that acquired relevance in this context is the view that thought experiments are operations with mental models (see Miščević 1992; Nersessian 2018). At the same time, attention partially shifted towards the question of what can be reliably asserted in counterfactual scenarios (see e.g. Williamson 2007; Häggqvist 2019). Nevertheless, the worry about how to learn about reality just by imagining remains, as well as Brown’s and Norton’s outposts in the search for an account of how knowledge or understanding of the world can be obtained from an investigation in which no new empirical information has been introduced.

This paper invokes Otto Neurath’s scientific utopianism to understand how novelty arises in thought experiments. We maintain that Neurath’s utopias can be understood as centerpieces of thought experiments. Indeed, not only Neurath’s logical empiricist philosophy but expressly his economic theorizing was strongly influenced by a pioneer in the philosophy of thought experiments, Ernst Mach (see Nemeth 2007). There are at least three disciplinary approaches to analyze the use of utopias as part of thought experiments. The first is by considering utopias in (thought) experiments in political philosophy (see e.g. Miščević 2018). The second is to consider that utopias are part of thought experiments because, as Catherine Elgin (2014) shows, many works of fiction and art can be so considered. This would emphasize the literary character of utopias. But utopias are not only pieces of political philosophy and of literature, as Neurath reminds us, so a third way is to conceive utopias as part of thought experiments in social science and technology. Each of these three disciplinary approaches can be carried out in different ways, according to the conception one assumes in regard to thought experiments. One can, for instance, consider that utopias are fictional narratives and so they can be understood as parts of thought experiments conceived in the context of mental modeling. This approach can be employed to understand the use of utopias in social science and technology and so to account for the uses of fiction in scientific contexts. However, although interesting and fruitful, such an outlook does not exclude the possibility of rationally reconstructing utopian thought experiments as arguments, as Norton proposes (Brendel 2018). Whatever the merits of conceiving of thought experiments as fictional narratives are, this paper focuses on the approach which rationally reconstructs thought experiments as arguments.

The next section presents Neurath’s utopianism. Section 3 briefly presents the debate between Brown and Norton and refines Sören Häggqvist’s model of thought experiments. By the application of our reformulation of Häggqvist’s model to one utopian and one dystopian example we will obtain insights on utopianism and on the argument view of thought experiments. Finally, Sect. 4 provides a third example and a Neurathian answer to the question as to how we can learn about the world just by using our imagination.

## Neurath’s Utopias

It is much more sensible to describe as utopias all orders of life which exist only in thought and image but not in reality, and not to use the word ‘utopias’ as expressing anything about their possibility or otherwise. Utopias could thus be set alongside the constructions of engineers, and one might with full justice call them social technological constructions (Neurath 1919/1979, 235).

Otto Neurath wrote these words in a text published over a century ago. His perspective is that social-scientific research can contribute to shaping the social order of the future. Accordingly, Neurath proposes that social science should inquire into different, imaginative social arrangements regardless of their practicability at the current technological and economic status and regardless of the likelihood of actualization of such new arrangements. By calling these exercises of creativity in social thought not only “conceived constructions” (Neurath 1922, 55), but also “*utopias*”, Neurath seeks to link this proposed scientific effort to the literary tradition of Thomas More, Étienne Cabet, Edward Bellamy, and others (Neurath 1919/1979, 236) in order to establish a *scientific utopianism* (Neurath 1944/1970). The task of utopianist scientific research, as Elisabeth Nemeth (1982/1991, 285–286) explains, is “to develop ‘groups of utopias’ and to make transparent the differences between these models in a ‘comparative utopistics’”.^2^

In Neurath’s case, any reproach of logical positivism as an apologetics of the (politically) given is entirely misplaced. Neurath believes that “utopias as social engineering constructions […] can make the mind flexible and free it from accidental notions” (Neurath 1919/1979, 239). Comparative utopistics can contribute to a broad debate on how to shape the social order of the future by presenting and comparing a variety of alternatives to the existing social order. Hence, Neurath projects that social scientific research will acquire a technological orientation towards new arrangements and institutions. The scientific study of utopias, Neurath continues, “would serve our young people better than traditional economic theory and sociology, which, being restricted to the past and the accidental present, were in no way able to cope with the tremendous upheavals of war and revolution” (Neurath 1919/1979, 240).

Contemporary technological and social-scientific efforts directed at establishing public policies notwithstanding, we are far from a Neurathian comparative utopistics. By stressing a continuity between technological, scientific, and literary or philosophical efforts to enhance social order, Neurath’s proposal concentrates efforts on social thought and highlights a methodological feature of the utopian tradition, that of taking into account the consequences that small social reforms entail in many parts of a community. Neurath’s scientific utopianism is neither restricted to comparing comprehensive social orders nor to full socialization, but amenable to piecemeal engineering as well. Some of his respective thought experiments compare how different wind directions (1917/2004, 317) or different actions on part of the gardener (1910/2004, 289) affect fruit trees and thus human well-being. As a more customary example, a utopian approach considers that installing a public health center can only represent an improvement in a community’s living conditions if accompanied by adjustments in the systems of sanitation, public transportation, and even in the electric power transmission—moreover, people must be informed of the potentials and limitations of the now-available medical care. As proposals of alternative social devices, utopias account for the fact that social situations are *Ballungen* (aggregates or clusters) of many interwoven aspects that can only be properly studied by a plurality of disciplines. In short, the work with utopias constitutes an interdisciplinary approach that looks into social problems from a wider perspective—even if in relation to a small community.

In considering comparative utopistics, that is, the establishment of a debate in which utopias are created and compared, we are dealing with investigations that use imaginary tools. A fruitful way to account for that is by characterizing utopias as centerpieces of thought experiments. As a matter of fact, such use is already discernible in Neurath’s perspective, although, obviously not in this contemporary terminology. Neurath claims that in utopias “individual elements […] are endowed with qualities which do not occur in real life or with real-life qualities, but in connections and in relation to transfers that so far have not occurred” (Neurath 1917/2004, 319–320). That is, scientific utopianism is to deal with counterfactual situations by elaborating groups of models which do not aim at a faithful representation of existing aspects of society but imagine counterfactual social systems in which for instance certain policies are implemented. Nevertheless, Neurath conceives a way to obtain knowledge of concrete situations, as he continues: “while possible worlds are thus admitted, it is also advisable to see to it that the system of models contains some [such models] from which conclusions about reality can be drawn” (Neurath 1917/2004, 320).^3^

## Utopias as the Linchpin of Thought Experiments

### Thought Experiments as Arguments

A divisive problem of contemporary philosophy is to give an epistemological account of thought experiments. How can their conclusions be justified? And if such a justification appeals to new data allegedly provided by thought experiments, then how can thought experiments produce new data? More generally, “how can we learn about reality […] just by thinking?” (Brown & Fehige 2017). As briefly stated in the introduction, the current debate revolves around two focal points. The first is James Robert Brown’s stance that thought experiments, in certain special cases, work similarly to (one account of) mathematical intuition by “allowing us to grasp the relevant universals” (Brown 1991/2011, 107). By accessing this Platonic realm, we allegedly have the possibility of obtaining intuited data from a plurality of sources and, hence, we are supposed to be able to justify conclusions from thought experiments, particularly in physics and philosophy (see Grundmann 2018 for a presentation and critical discussion).

The second is John Norton’s empiricist position of taking thought experiments to be arguments. By “reducing” thought experiments to arguments, he purports to explain the justification of contingent propositions which a successful thought experiment appears to provide. As Elke Brendel (2018) explains, this claim of “reducibility” can have at least five meanings: (i) that thought experiments are type-identical with arguments; (ii) that thought experiments can be rationally reconstructed as arguments that yield the same outcome; (iii) that thought experiments have the same epistemic power as arguments; (iv) that the conduction of a thought experiment is the execution of an argument; (v) that the outcomes of thought experiments result from procedures that preserve truth or probability, as in arguments. Based on Brendel’s and Gendler’s (2000, 34–39) further analyses of the reconstruction thesis, we can identify different interpretations of the reconstruction thesis (ii): one that includes the claim (ii-a) that the justificatory force provided by a thought experiment stems from and can only be explained by the justificatory force provided by a reconstructed non-thought-experimental argument. Hence, “a thought experiment is a ‘reliable mode of inquiry’ only if the argument into which it can be reconstructed justifies its conclusion” (Brendel 2018, 283) and, therefore, thought experiments can arguably be eliminated. Another interpretation of (ii) is limited to (ii-b) that a thought experiment can be reconstructed as a (non-thought-experimental) argument that yields the same outcome, regardless of justificatory force. For the purpose of this paper, we will focus mainly on the rather weak thesis (ii-b).^4^ We will also draw on a weaker variant of the execution thesis (iv), namely that the conduction of a thought experiment can be reconstructed and understood as the conduction of an argument. We do not intend further commitment to stronger theses, such as the identity claim (i) that thought experiments are identical to arguments.

While Brown’s Platonism accounts for the discovery of new data and for the justification of conclusions obtained by a thought experiment by means of abstract intuition, Norton’s (1996; 2004a) proposal focuses on the reconstruction of definitions, of descriptions of (possible) experience, and of other presuppositions of a thought experiment. They all enter a deductive or inductive argument to be reconstructed from the thought experiment as premises. The conclusion of the argument then becomes justified relative to the premises (and the rules of inference). The empiricist tone of Norton’s argument view seems to be most suitable for engaging thought experiments involving Neurath’s utopias. Accordingly, we will characterize Neurath’s utopias as centerpieces of arguments and, as such, as parts of processes of reasoning in Sect. 3. As our discussion in Sect. 4 will show, the further exploration and reorganization of theoretical structures, as well as the new descriptions of previously known data, can lead thought experimenters to establish new knowledge. Freshly devised or already held beliefs are justified by inferring them from known or hypothetical premises. Section 4 will also convey Neurath’s claim that the attitude and practice of scientific utopianism facilitates genuine discoveries.

We will build upon an important development close to Norton’s weaker reconstruction thesis (ii-b) of thought experiments, obtained by Sören Häggqvist (2009). His perspective emphasizes similarities of thought experiments with “actual” experiments for tests of hypotheses and theories—in contrast to other uses such as thought experiments as illustrations or explanations. In Häggqvist’s view, many thought experiments can be regimented in the following schema (Häggqvist 2009, 63).
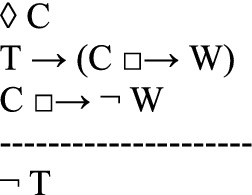
in which *C* is the counterfactual scenario that the thought experiment describes as possible (first line); *T*, the theory to be tested, implies that if *C* were the case, then a state of affairs *W* would be the case (second line). However, the thought experiment shows that if *C* were the case, then it would not be the case that *W* (third line). Since *T* is committed to *W*, and since *W* is shown to be false in the counterfactual scenario, the thought experiment concludes that *T* is false (fourth line). Instances of this argument schema are valid arguments for usual semantics of counterfactuals.

It is common that defendants of a theory argue against a thought experiment. Häggqvist’s schema takes into account the argumentative possibility of rejecting the thought experiment. That is, the proponents of a thought experiment argue that the theory *T* is false. But their opponents, of course, might wish to save the theory *T*. So they may argue that 
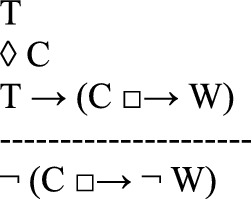
concluding that it is not the case that if *C* were the case, then *non-W* would be the case. They may also argue that 
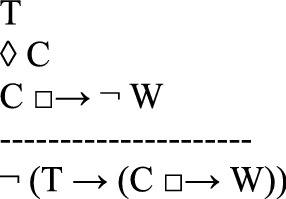
the theory *T* is not committed to (*W*, if *C* were the case). Another possibility, still according to Häggqvist (2009, 65–68), is to claim that the counterfactual state *C* is not possible:
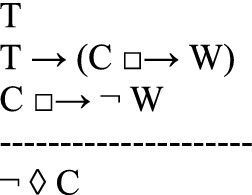


One way of looking at Häggqvist’s model for thought experiments is that each thought experiment is linked to four possible types of arguments. Indeed, since the argument view would suggest that each thought experiment is linked to exactly one argument type, Häggqvist (2009, 61) concludes that the identity thesis (i) of the argument view of thought experiments is not quite correct.^5^ However, a one-to-one correspondence between thought experiments and argument types could be upheld by slightly reformulating Häggqvist’s model for thought experiments.^6^ Moreover, we maintain that the reformulation adequately complies with many uses of thought experiments, especially with the open-ended discussions of utopias and dystopias.

Häggqvist expounds how one thought experiment can be reconstructed as one of four argument types, each of which negates one of the claims T, ◊ C, T → (C □ → W)*,* or C □ → $\neg$W. We propose another way of putting this, namely that one thought experiment can be reconstructed as one (meta-)argument to the effect that the set of claims {T, ◊ C, T → (C □ → W), C □ → $\neg$ W} is inconsistent. The meta-argument for inconsistency can be specified as an argument with premises and a contradictory conclusion (see also Sect. 4.3 for a specific example):
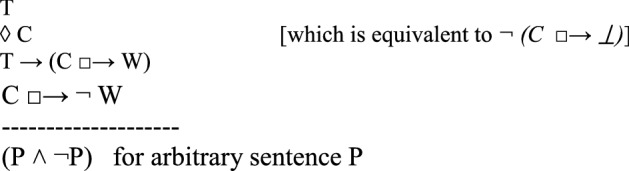


Since﻿ an inconsistency allows the inference of any sentence and its negation, this poses a problem. After all, we do not want to collapse the system of our descriptive beliefs or of our evaluations.^7^ From this perspective, a thought experiment exhibits an inconsistency and urges the addressee to reject at least one of the four claims *T*, *◊ C*, *T → (C □ → W)*, or *C □ → *$\neg$*W*.^8^ However, the decision which of the four claims to discard is not logically determined by the thought experiment.

Our approach is partially in line with Gendler’s (2000, 59–62) criticism of Norton (and Brown) insofar as both of them sometimes pay insufficient attention to different alternatives how to resolve the inconsistency revealed by a thought experiment. In their discussions of the paradigm example of Galileo’s falling objects and perhaps also in the case of Russell’s antinomy Norton and Brown typically present only one solution to the inconsistency although “the reductio tells us that something is wrong […] but it does not tell us what is wrong” (Gendler 2000, 62). Notwithstanding some amendable presentations, Norton (2004a, 59) (and arguably Brown) fully acknowledge that in reductio arguments, “[i]n principle, any of the premises of the argument—tacit or explicit—may be taken to have been refuted” and embrace this as a property of the argument view. Merely relying on intuition can be misleading; further arguments are necessary to support a decision how to resolve a revealed inconsistency. By contrast, Gendler’s constructivism purports to offer an account of how the scenario of a thought experiment correctly guides the contemplation of a constructively participating reader to specific insights on how to reconfigure her internal conceptual space, which premise to drop, or how to adapt her inconsistent beliefs. Yet, if the epistemic force of this guide to the correct response cannot be reconstructed as an argument, it is not clear why it would be credible or reliable (see Gendler 2000, 33–63; Norton 2004a; 58–59; 2004b, 1148–1149). Moreover, from Neurath’s anti-Kantian perspective, Gendler’s constructivism would likely have to be rejected for not being strictly empiricist. Accordingly, Gendler (2000, 151) refers to Norton’s argument view as “immoderate empiricism”.

In any case, we would like to highlight one consensus between Gendler, Norton, and our reformulation of Häggqvist’s model: a thought experiment does not logically determine a unique course of action. We maintain that this openness adequately reflects the ubiquitous disagreements about the conclusions to be drawn from many thought experiments in literature, philosophy, and the sciences. In fact, many thought experiments elicit quite different reactions to the revealed inconsistency. While Häggqvist reconstructs the four types of reactions as *arguments*, our perspective treats them as four types of *decisions* in light of one (meta-)argument. All our examples could easily be reconstructed using Häggqvists original perspective as well; Häggqvist (2009, 65) even uses the phrase four “ways of resolving the inconsistency”. Yet, it is very much in Neurath’s spirit to accentuate the indispensable and oftentimes hidden role of decisions in science. In Sects. 3.3, 3.4, and 4.3, we exemplify how the use of utopias in thought experiments fits our reformulation of Häggqvist’s model.

### Neurath and Thought Experiments

As stated above, Neurath’s scientific utopianism proposes that utopian thought experiments can and should be used in social scientific research and policy debate. We will apply our reformulation of Häggqvist’s model of thought experiments to utopias and dystopias. Firstly, this demonstrates the fruitfulness of the model; secondly, we gain insights on Neurath’s utopianism.^9^

It is important to remark that a utopia is to be regarded as the linchpin of a thought experiment, albeit not the entire thought experiment. Utopias are merely presentations of counterfactual (and hence logically possible) situations. A thought experiment considers a usually stronger, e.g. physical, claim of possibility for said utopia (◊*C* in Häggqvist’s formulation), in an inquiry together with other claims involving this stronger sense of possibility, such as *T*, *T → (C □ → W)*, and *C □ → *$\neg$*W*. It is the *combination* of these three elements *and* ◊*C* which amounts to a full thought experiment in Häggqvist’s model and in our reformulation thereof. Besides, while utopias can be used in thought experiments, that is not their only application, and not even the main or most frequent context in which people reflect about them.

Oftentimes, utopists try to show particular outcomes of implementing plans and that the outcome is *not* what our prevalent understanding of society regards as a typical or necessary consequence of *C*. Therefore, many utopists not only present ◊*C*, but also argue that *C* □ → $\neg$*W*. More precisely, utopists illustrate that if a counterfactual situation *C* were the case, it would have some particular state of affairs as an outcome, part of which is described by $\neg$*W*. However, especially in literary utopias, the likelihood of the described outcome and what to conclude from the general understanding of society are sketchy at best.

Utopian narratives do not typically include a presentation of *T*, that is, of our (theoretical or common) understanding of how social arrangements work. To mention some famous examples, Thomas More does not explain the underlying theories of state and economy of his time, neither does Aldous Huxley enter into much detail of the social science behind the *Brave New World* (see Berneri 1950/1971). In addition, utopian narratives do not usually present the implication *T* → (*C* □ → *W*) that the assumed theory entails that, if the utopian scheme were the case, some particular state of affairs would follow. These two implicit elements (*T*, as well as the implication *T* → (*C* □ → *W*)) are fundamental for readers to have an experience of the utopia as part of a thought experiment. In short, utopias can be regarded as works of art, and then their presuppositions may or may not be of consequence, but they can also be regarded as pieces of social philosophy or of social science (see Vieira 2010). In this latter use, utopias are centerpieces of thought experiments—and according to Norton’s argument view, these thought experiments can be reconstructed as arguments.^10^

The role of utopias in thought experiments will become clearer with the following examples: Sect. 3.3 presents the use of a Neurathian utopia in a thought experiment in its simplest form. A more sophisticated version will come up in Sect. 4. The dystopian example in Sect. 3.4 showcases the advantages of our reformulation of Häggqvist’s model and hints at some intricacies of the role of evaluations in thought experiments.

### Example U: Neurathian Utopia, Simple Case

Our first example reconstructs the use of a Neurathian utopia in a thought experiment as an argument that makes it explicit that the set {T_U_, ◊C_U_, T_U_ → (C_U_ □ → W_U_), C_U_ □ → $\neg$W_U_} is inconsistent. An explication of the four components T_U_, ◊C_U_, T_U_ → (C_U_ □ → W_U_), and C_U_ □ → $\neg$W_U_ will be sufficient to apprehend the inconsistency.

T_U_: T_U_ is the political and economic background theory Neurath perceives to be dominant at his time. According to T_U_, private property of the means of production and capitalistic profit calculation bring about optimal overall outcome. For the sake of argument, we follow Neurath in roughly identifying such a capitalistic state of affairs with the actual situation A in his time.

◊C_U_: The counterfactual situation C_U_ is one of Neurath’s utopias, i.e. an outline of one of several possible ways of socialization (see e.g. Neurath 1920/2004, 347–356). Subsequently, the typical outcome of this communizing or socializing of the means of production is scientifically investigated.^11^ Neurath’s scientific utopias stand out due to their comparatively elaborated analysis of the outcome in utopian social orders. From Neurath’s perspective, the inquiry of the outcome has to encompass many different, irreducible dimensions, which he called “conditions of life”. They may for instance include housing conditions, infant mortality, illiteracy, leisure hours, likelihood of illness, hygiene, the use of radio sets, opportunities for amusement, and nutrition. Prospected effects on these “conditions of life” are investigated and presented in Neurath’s “universal statistics” (Neurath 1921/2004).

T_U_ → (C_U_ □ → W_U_): Let W_U_ be the sentence that any typical overall outcome of adopting the social order C_U_ is inferior to (the predicted future outcome of) the actual situation A. Then it holds trivially that *if private property of the means of production brings about optimal overall outcome, then (if the socialization schema C*_*U*_* were implemented then its typical overall outcome would be inferior to the capitalistic A)*.

C_U_ □ →$\neg$W_U_: A careful study of C_U_ along many dimensions of welfare shows that living conditions in the utopia are overall not inferior to living conditions in A. Note that Neurath insists on a subjective element here. The many dimensions of welfare should not be weighed to allow for an algorithmic decision, but rather reflected and deliberated upon in a democratic process (Nemeth 2019; Neurath 1996, 249–261; O’Neill 2007, 188–195). However, the appraisal of a vast majority preferring the overall outcome of reasonable planning schemata to the overall outcome of capitalism seemed obvious to Neurath.^12^

While logically all other possibilities to dissolve the inconsistency of {T_U_, ◊C_U_, T_U_ → (C_U_ □ → W_U_), C_U_ → $\neg$W_U_} are available, example U neatly represents the standard case in Häggqvist’s schema, i.e. an attack on the theory T_U_. Part of T_U_ is the sentence that capitalism is the most “efficient” and welfare-benefiting arrangement, which Neurath challenged in imaginary and also in real-life experiments during his time in the Munich Soviet Republics. One main function of Neurath’s scientific utopias is to challenge and ultimately reject those parts of the political and economic theory T_U_ which maintain that private property of the means of production and capitalistic profit calculation bring about optimal overall outcome. According to Neurath, the ensuing political consequence would be to implement the planning schema C_U_ (while continually considering alternative planning schemata): “He who deduces an order which provides more pleasure than our present one becomes a scientific utopian. His views can stimulate himself or others to actualize that order” (Neurath 1911/1998, 517).

Thought experiment U does not establish an unconditional imperative to implement socialism—arguably, neither does any other thought experiment in regard to any topic or socio-economic order.^13^ The logical structure of thought experiment U does not even imply which specific part of T_U_ to drop or whether T_U_ has to be changed in the first place. Alternatively, the truth of any other premise can be challenged, with the empirical claim *C*_*U*_* □ → *$\neg$*W*_*U*_ being a plausible candidate for a false premise; i.e. one could argue that the living conditions in the socialist utopia would actually be inferior to the actual living conditions. Our next example explores different options of how to deal with the inconsistency revealed by dystopian thought experiments.

### Example D: Dystopian Novels

Dystopian novels contain or trigger another group of thought experiments.^14^ The epistemic value of literary dystopias is comparable to that of scientific utopias as Cunha (2015, 2018) has argued for the examples of Huxley’s *Brave New World* and Zamyatin’s *We.* Other famous examples include Orwell’s *Animal Farm*, Rand’s *Anthem* or Le Guin’s *The Dispossessed*. Typically, a dystopian novel starts by outlining a counterfactual social system which prima facie appears desirable. However, as the story unfolds, the protagonists face hitherto neglected aspects of that social system. These unintended developments of the dystopian system urge the reader to reevaluate her initially positive appraisal of the situation.^15^

Our reformulation of Häggqvist’s model for thought experiments allows for a viable reconstruction of the typical overarching argument of a dystopian novel as an argument that makes the inconsistency of the set {T_D_, ◊C_D_, T_D_ → (C_D_ □ → W_D_), C_D_ □ → $\neg$W_D_} explicit. Our perspective remains faithful to the startling experience which some dystopian novels provoke. In many dystopian novels, the author does not prescribe an obvious conclusion but stimulates reflection and discussion. The reader realizes that her beliefs or evaluations are challenged, but it is up to her which ones to adapt or discard. We will discuss reactions to dystopian novels along Häggqvist’s four options, but let us start with a brief reconstruction of the four premises of the argument:

T_D_: The background theory T_D_ includes the sentence that certain technological, legislative, organizational, or ethical principles p_1_, p_2_, p_3_ and their typical consequences are overall desirable (according to a given group of persons).^16^ In Huxley’s *Brave New World*, to use a widely famous example, some readers and arguably many of the fictional inhabitants may initially hold such a background theory T_D_, including the belief that a better society would be obtained by implementing scientific techniques, such as genetic engineering, psychological conditioning, and treatments with psychoactive drugs. A resulting society is regarded as “better” because it warrants that every citizen has a job, social life, and entertainment—and also that everyone is happy about it. If a reader neither contemplates any such background theory T_D_ nor interprets the fictional characters as contemplating parts of T_D_, thought experiment D does not enrich her perusal of the novel (see Huxley 1932/2006).

◊C_D_: In the counterfactual, but possible situation C_D_ the principles p_1_, p_2_, p_3_ are implemented in the social order. Huxley’s imaginary *Brave New World* presents a whole aggregate of many different kinds of technology. From videophones and flying machines to educational, psychological and pharmacological technology—besides the pivotal genetic engineering.

T_D_ → (C_D_ □ → W_D_): Let W_D_ be the sentence that any typical overall outcome of implementing the principles p_1_, p_2_, p_3_ is desirable. Then the full sentence states: *If the typical consequences of* *p*_1_, *p*_2_, *p*_3_ *are desirable, then (if* *p*_1_, *p*_2_, *p*_3_ *were implemented, then any typical overall outcome of p*_*1*_*, p*_*2*_*, p*_*3*_* would be desirable).* Note that T_D_ implies W_D_, hence T_D_ also implies C_D_ □ → W_D_ for usual semantics of counterfactuals. So, if scientific techniques in social planning produce a desirable society, then *Brave New World*’s society would be desirable.

C_D_ □ → $\neg$W_D_: As the dystopian novel unfolds, a hitherto unheeded typical outcome O_D_ of the counterfactual situation C_D_ is revealed. Taking into account the hitherto unforeseen developments warrants the claim that, surprisingly, the typical outcome O_D_ would not be desirable. Therefore, if the principles p_1_, p_2_, p_3_ were implemented in the social order, it would not be the case that all the typical outcomes would be desirable. Huxley’s story depicts most main characters as unhappy about the outcome of their situation. They genuinely experience great suffering. The reader might interpret some protagonists, particularly Helmholtz Watson, as changing their appraisal of the desirability of the brave new world (for a given group of persons) over the course of the novel. On top of that, many readers might ponder whether they themselves would like to live in that world and whether their friends and neighbors would enjoy it. Arguably, the novel at the very least indicates that for some fictional and for some real persons, on closer scrutiny such a society is not desirable at all.^17^

Several strategies for resolving the ensuing inconsistency of the set {T_D_, ◊C_D_, T_D_ → (C_D_ □ → W_D_), C_D_ □ → $\neg$W_D_} are possible. Ultimately, this final step involves a decision. Such a decision “is no longer the task of science, strictly speaking, which points out possibilities and determines the facts of the past, present, and future” (Neurath 1921/2004, 370). Indeed, following Neurath, we could award his label of *pseudorationalism* to the sentiment that such decisions can ultimately be justified by logical or empirical means in each case (see Neurath 1913/1983; 1945/2004).^18^ The inconsistency brought about by our reformulation of Häggqvist’s model for thought experiments resembles the inconsistency brought about by experiments. Logic does not compel the experimenter to a particular decision (see also Duhem 1906/2007). One can either reject a part of the theory, or one of the boundary conditions, or the auxiliary theories, or the protocol describing the outcome of the experiment. Among the possible decisions for resolving the inconsistency in our dystopian example D are the following:

1.a. Challenge T_D_. Contrary to a first impression, the technological, legislative, organizational, or ethical principles p_1_, p_2_, p_3_ and their typical consequences are not overall desirable. Similarly as for scientific and philosophical thought experiments, challenging the underlying theory usually seems to be the intention of dystopian novels. Huxley’s *Brave New World* can reasonably be read this way as the author himself suggests (Huxley 1958/2006; also see Huxley 1932/2006).

1.b De-ontologize T_D_. The motto of this solution is “Let justice be done, though the world perish.” If a champion of the technological, legislative, organizational, or ethical principles p_1_, p_2_, p_3_ accepts the argument that their consequences are undesirable, she can revert to an immunizing strategy. The new T_D_* only states that p_1_, p_2_, p_3_ are desirable—no matter what the consequences are.

2. Shrug off C_D_. The counterfactual social situation C_D_ described in the dystopian novel can be judged as impossible or irrelevant. If those authors who impose stricter standards of conceivability, possibility, or relevance for applied contexts are correct,^19^ then arguments against conceivability, possibility, or relevance are particularly pressing for utopianism.

Instructive examples of “shrugging off” can be found in the thought experiments of Ludwig Mises, Neurath’s main intellectual sparring partner in the socialist calculation debates and in comparative economic systems (Linsbichler 2015; 2022a). Mises (1949/1998, 202) and other Austrian economists^20^ regularly employ thought experiments involving “imaginary constructions to which nothing corresponds in reality” as “an indispensable tool of thinking” and ultimately conclude that social states depicted in these imaginary constructions are impossible.^21^

3.a. Exceptional case. One could challenge whether the dystopian novel actually portrays a typical development or whether the main characters are representative of any relevant groups of persons in the real world. If O_D_ is not a typical outcome of implementing p_1_, p_2_, p_3_, then it does not matter whether O_D_ is undesirable. Note however that even relatively improbable consequences might be crucial if they are extremely undesirable.

3.b. Sugarcoat O_D_. Häggqvist (2009, 66) refers to this solution as “biting the bullet”, i.e. accepting and embracing the typical outcome O_D_ in spite of some aspects (perhaps not only initially) being perceived as negative. Although this seems to be an ad hoc solution, the overall desirability of the typical outcome O_D_ can certainly be reconsidered. Even after becoming aware of all the drawbacks of a *Brave New World,* some readers might reason that a guaranteed job, sexual freedom, and socially accepted drugs that cause no hangover do after all outweigh permanent shallowness. That is, readers might not empathize with the main characters but instead agree with Mustapha Mond, the only character in the novel who clearly understands the trade-off and after careful consideration unflinchingly bites the bullet that “community, identity, and stability” are worthwhile sacrificing art and individuality. One could perhaps interpret the ending of Orwell’s *1984* as an even more drastic example of sugarcoating: although most readers likely find the fictitious totalitarian state Oceania atrocious and its leadership symbol Big Brother repulsive, after “re-education” in the Ministry of Love, (disputedly) the protagonist Winston loves Big Brother.

To be clear, what is to be reconsidered is the descriptive sentences describing the subjective desires and preferences of a given group of people.

Hitherto, we have reconstructed possible resolutions of the inconsistency of {T_D_, ◊C_D_, T_D_ → (C_D_ □ → W_D_), C_D_ □ → $\neg$W_D_} from the perspective of a social scientist who analyses a dystopian novel and theorizes about how a given group of persons evaluates the principles p_1_, p_2_, p_3_ and their typical outcome O_D_. The dystopian thought experiment presents an inconsistency that urges the social scientist to adapt her beliefs regarding valuations, i.e. reconsider the descriptive sentences about subjective desires and preferences of a given group of people (solutions 1.a, 1.b, and 3.b). Whereas this perspective fits the format of logical derivations in arguments reconstructed from the thought experiment, perhaps the format of a novel or of a filigreed presentation of a thought experiment is more akin to a different perspective: instead of challenging beliefs about other persons’ evaluations, a dystopian novel might be primarily viewed as a challenge to the reader’s own evaluations. From this perspective, the dystopian novel is not primarily concerned with consistency of beliefs but prompts the reader to reflect upon inconsistencies in her evaluations—and eventually re-calibrate her preferences and desires so as to act in a certain way.

## Is Neurath a Good Empiricist?

### The Tension between Platonism and (Neurath’s) Scientific Utopianism

So far, we argued that Neurath’s utopias can be considered as linchpins of thought experiments, and we exemplified how Häggqvist’s refined model can be used in order to reconstruct the use of a utopia in an argument. In Sect. 4, we argue that Neurath’s scientific utopianism avoids Platonism and helps to comprehend novelty in the argument view of thought experiments.

Norton’s argument view faces criticism for purportedly not being able to account for justifications of empirical beliefs and genuine discoveries obtained by thought experiments (see e.g. French and Murphy 2021, Sect. 3). After all, if thought experiments are merely arguments, the conclusions obtained are already contained in the premises (and the rules of inference).^22^ Since the possibility of novel justifications, connections, and discoveries is a crucial component of Neurath’s scientific utopianism, there is a potential problem: Neurath’s strict empiricism commits him to a position very close to the argument view but it is prima facie questionable whether the argument view can provide novelty as required.^23^

Admittedly, there are quite mundane ways in which Neurath’s scientific utopianism can expand scientific knowledge. “New” knowledge, i.e. knowledge contained in the premises (and the rules of inference) but hitherto not recognized, can be produced either by conceptual exploration or by considering new, possible boundary conditions and applying the latest social scientific theory to them. Ideally, this is what happens when the conditions of life in a given utopia are explored and depicted. While “new” knowledge generated that way may sometimes be relevant for its own sake, it is not really what scientific utopianism aims for, but merely a means, an intermediate step, before arriving at fundamentally new insights.

Apart from these mundane ways, how can the use of utopias in thought experiments foster fundamentally new discoveries? Trying to make sense of Neurath’s quest for novelty, we are cast back to the contemporary dispute about the epistemology of thought experiments touched upon in Sect. 3.1.

Brown’s Platonistic view of thought experiments explains the obtainment of fundamentally new insights quite straightforwardly. According to Brown, at least some thought experiments grant access to “objects whose nature, as normally conceived, places them beyond the reach of the better understood means of human cognition (e.g., sense perception and the like)” (Benacerraf 1973, 667–668). Consequently, these special thought experiments trigger experience of a special kind and thereby fundamentally new insights. The Platonistic assumptions underlying Brown’s account of the discovery and justification of new knowledge by means of thought experiments are most controversial from an empiricist stance. How can a strict empiricist like Neurath account for new insights through thought experiments?

### The Argument View, Utopianism, and Novelty

Whatever the merits of the Platonistic view are, Neurath’s strictly empiristic oeuvre obviously conflicts with Brown’s (and any other) Platonism since empiricism rejects the possibility of acquiring empirical knowledge without sense perception. As a good empiricist, Neurath’s use of utopias in thought experiments should be explicable in terms of Norton’s argument view or some other strictly empiricist view.^24^ Over and above clarifying Neurath’s ideas, such an explication contributes to a more comprehensive view of novelty in the argument view of thought experiments, acknowledging that utopias are hybrid imaginary constructions of literature, science, and philosophy. Indeed, scientific utopianism contemplates the quest for novelty within the argument view in three distinct (yet connected) ways, all of which Neurath already contemplated: (I) Dealing with utopias and thought experiments on a regular basis increases creativity and inventiveness. (II) Particular ways of presenting knowledge facilitate scientific discovery and social progress. (III) The use of utopias in thought experiments can prompt conceptual change and hence allows access to new phenomena.

Our example N in Sect. 4.3 below highlights how Norton’s argument view elucidates a case of (III). In particular, Häggqvist’s model of thought experiments and our reformulation of it ideally suit the context of testing claims (and definitions of concepts in these claims). In comparison, (I) and (II) are not directly concerned with testing or with epistemic novelty, but rather with psychological prerequisites of quests for novelty. In-depth studies of (I) and (II) would require understanding thought experiments by means of other approaches than Norton’s and Häggqvist’s.^25^ The main purpose of this section is firstly to indicate Neurath’s awareness of (I) and (II) and secondly to clarify that contrary to some misrepresentations, Norton’s argument view is compatible with in-depth studies of (I) and (II).

(I) Scientific utopianism plays an eminent role in social engineering. While scientific mechanics imagine new machines, some of which will be materialized by mechanical engineers, “utopianists” contribute to social engineering. Neurath conjectures that the gravest obstacle to technological and social improvements might be limitations in “finding and handling possible solutions” (Neurath 1944/1970, 31). As a remedy, scientific utopianism encourages involvement with utopias, thought experiments, and counterfactual scenarios in general as a means to instill a habit of constantly imagining alternatives to the status quo. This experimental habit is ideal-typically manifested in utopian and dystopian novelists. For social scientific purposes, such a “sense of possibility” (Robert Musil) should be combined with the analytic attitude to investigate and compare outcomes scientifically.

We construe Neurath as maintaining that dealing with utopias and thought experiments on a regular basis increases creativity and inventiveness, both among social scientists and among a more general public. Ultimately, utopianism can help to bring about groundbreaking technological inventions, improved social orders, new scientific knowledge, and a willingness to embrace these novelties (Neurath 1944/1970). Expressed in terms of the arguments reconstructible from the thought experiments, an attitude and practice of scientific utopianism motivates searches, creations, and discoveries of new potential premises, new arguments, new possible conclusions, new descriptions of possibly new counterfactual scenarios, and new hypotheses about properties of hypothetical scenarios. To the extent to which new arguments are devised or premises can be further justified, scientific utopianism also induces new justifications.

(II) Neurath is fully aware that the way knowledge is presented impacts the ability to grasp it, reason independently about it, and discover new connections in it. Regarding his activities in picture statistics and museum design, the “challenge was to create special tools for discovering and revealing social facts” so that laymen but also scientists “could learn to look at social issues in a new way” (Nemeth 2019, 126). Utopias and thought experiments can be considered tools with a similar purpose.^26^

However, the idea that inventors and audience of thought experiments are, perhaps due to some psychological mechanism, more likely to elicit scientific discoveries and social progress prima facie seems to conflict with Norton’s argument view. If all that matters about a thought experiment is the logical structure of the reconstructible argument type, then picturesque, emotional, or performative aspects of a thought experiment are just decorative accessories. Nonetheless, a thought experiment can be extremely convincing or stimulating for a particular crowd just because of its artful presentation and in spite of a flawed logical structure of the reconstructible underlying argument. Neurath distinctly affirms the importance of the mode of presentation, as he notices that “with their attractive descriptions, horror stories and social poetry in the form of novels the early utopians prepared people’s emotions and their will to shape their lives deliberately” (Neurath 1920/2004, 394). At a first glance, this seems to contradict the argument view.

Contrary to some of its portrayals (see e.g. the otherwise laudable Brown & Fehige 2017, Sect. 3.2; and Islas Mondragón 2020, 60–62), however, the argument view of thought experiments is not restricted to the more famous *reconstruction thesis* that every thought experiment can be logically reconstructed as an argument. Norton fully acknowledges the importance of presentation and also advocates the *execution thesis* according to which “the actual conduct of a thought experiment consists of the execution of an argument”, even if in disguised form (Norton 2004b, 1142–1143). For our purposes, it is even sufficient to settle for being able to reconstruct or understand the execution of a thought experiment as the execution of an argument. By *understanding* the execution of a thought experiment as the execution of an argument, we are able to investigate not only the logical structure of an argument, but also rhetoric, performative, psychological, or pragmatic aspects of thought experiments just like in the case of (other) arguments. Even though in principle one cannot justify or learn more from a thought experiment than from its associated argument, oftentimes what people actually do discover, learn, or believe to learn depends on how the argument is presented (see also Brendel 2018, 283).^27^ An argument can successfully exhibit an inconsistency but whether an audience is convinced of the inconsistency and is motivated to readjust its beliefs depends on the presentation of the argument. Due to the somewhat neglected execution thesis, the argument view can account for the claim that the rhetoric of a thought experiment might play a psychological role in its inventor or audience.

A related but more cognitivistic demur to the argument view states that some thought experiments yield conclusions by mobilizing further cognitive resources including prior empirical knowledge (see e.g. Davies 2018; Elgin 2014; Miščević 2018; Nersessian 2018). These resources are activated when the (mental) model of the counterfactual scenario is appropriately manipulated. So far, this is perfectly compatible with the reconstruction thesis of the argument view. In an attempt to construct a sound argument, attempted arguments may be supplemented by additional premises and additional rules of inference. In some cases, recognizing that these additional premises are necessary to arrive at the desired conclusion constitutes a valuable insight. The reconstruction of a thought experiment can make implicit assumptions explicit, as already Mach (1906/1917) indicated.

However, proponents of an inflationist view of thought experiments maintain that some of these further cognitive resources and some manipulations of (mental) models cannot be articulated explicitly in propositional form (Davies 2018). Hence, purportedly no adequate reconstruction in deductive, inductive, or abductive argument form is possible. Defenders of the argument view can reply that communication about a thought experiment almost always assumes propositional form when the counterfactual scenario, the background theory, and alleged outcomes are described. These descriptions can be construed as premises and a desired conclusion of an argument. Norton’s reconstruction thesis then prompts an assessment whether the desired conclusion is justified by the premises and which rules of inference are deployed in the justification:

That is not to say that all thought experiments are instances of perfect deductive or inductive inference. Thought experiments can be bungled, just as arguments can. Rather, when we evaluate thought experiments as epistemological devices, the point is that we should evaluate them as arguments. A good thought experiment is a good argument; a bad thought experiment is a bad argument (Norton 1996, 335; see also Norton 2004a, 58–59).

If some intermediate steps in the thought experiment cannot be adequately articulated in propositional form, the reconstruction of the thought experiment will turn out as an argument with gaps. The desired conclusion might be a fascinating hypothesis worth further exploration but remains unjustified by the premises—given a notion of justification that is somehow explicable as rule-following.^28^ The execution thesis then invites us to study how and why the desired conclusion was discovered or intuitively regarded plausible in spite of the lack of justification.^29^

(III) Finally, we encounter the most fundamental variant of Neurath’s quest for novelty via utopianism. Inspired by Mach’s ideas in physics, Neurath aims at a reconstruction of the conceptual basis of the social sciences (see Nemeth 2007; 2013). If successful, this facilitates an expansion of experience, access to new phenomena, and innovative insights both for a scientific discipline and for our everyday outlook on the social world.^30^

Neurath (1935/1987, 103) perceived a “deep-seated false orientation” of economics. He proposed a revision of an overly constricted subject matter of economics by extending its “conceptual structure” (Neurath 1917/2004, title). Among other things, Neurath advocates a recollection and advancement of the broad notions of “wealth” and “happiness” common to classical economists and Carl Menger (Neurath 1911/1998, 500), but foreign to the *homo economicus*. Accordingly, Neurath has been portrayed as consciously attempting to “*re*gain a scientific object” (Nemeth 1999, title, emphasis added). Partially anticipating ideas of Kuhn, Gendler, and Hacking, he considers utopias and thought experiments as particularly apt for his aim to “dislodge a person from a certain way of describing the world” (Hacking 1992, 307).

Similar accusations that economics has unduly narrowed its conceptual basis and subject matter have been widely shared in public discourse and in academia in recent years. Not only are attempts to broaden the conceptual basis of economics a highly controversial topic in the twenty-first century; likewise, the historical origins of utopianism can be read as primarily concerned with an extension of possible thought and argument. Thomas More’s eponymous *Utopia* has been interpreted as aiming for a broadening of the discursive space for discussions about desirable social states. Arnswald (2019) argues that More’s objective was not to construct an ideal society, but to provoke critical thinking and trigger discussions about the status quo and alternatives to it in a wider space of possible discourse. Furthermore, Arnswald explicitly pursues the connection between utopias and thought experiments in Mach and Neurath. In the next section, we use our reformulation of Häggqvist’s model for thought experiments to rationally reconstruct how Neurath employs utopias as parts of thought experiments in order to broaden the conceptual structure of economics.

### Example N: Neurathian Utopia, Harder Case

For a few decades now, debates on how to compare social orders have been gaining currency again. In particular, the notions of well-being, welfare, and flourishing have been disputed with regards to their definitions and to the methods of their assessment. While Neurath pioneered the propagation of irreducibly multidimensional notions of well-being, it was Amartya Sen who played a key role in making them highly topical, particularly in poverty research (see Leßmann 2007). More generally speaking, however, gross national product still plays a predominant role as a single criterion in comparisons of social states. Our example N in this section reconstructs how Neurath challenges the prevalence of a single dimension of comparison.

In a simple application of utopias, like example U in Sect. 3.3, different social situations are compared and ranked. A (simplified) outcome could be that planning schema C_2_ is superior to capitalism, which is in turn superior to planning schema C_1_. By contrast, example N investigates the very notion of “superiority” underlying such comparisons. As Neurath (1911/1998, 503–504) stresses, utopianism as an inquiry in the theory of wealth appertains to theoretical, not practical political economy.

For a start, consider the following analogue of these different perspectives in another context: when Neurath (1939, 43–62) outlines trends towards modernization, he admittedly compares different countries and their historical development. However, he also reflects upon different aspects of “modernization” and demonstrates that these aspects are not always correlated. For instance, comparing France and Germany between 1914 and 1937, Neurath shows that, relatively speaking, France’s modernization manifested itself much more strongly in terms of automobiles per capita as opposed to Germany’s more rapid modernization in terms of telephones per capita (Neurath 1939, 59). In his words,

[i]f a country is more ‘modern’ in one field, it is not necessarily ‘modern’ in all the others. A general or average ‘index of modernity’ conceals certain peculiarities which are important not only in technologically appraising single countries but in appraising the whole process of modernization (Neurath 1939, 59).

Transcending “modernization”, any useful comparison between two social states should, according to Neurath, recognize and incorporate different dimensions. This becomes even more crucial when a comparison is taken as a basis for a decision which social state to strive for to improve human well-being. We reconstruct such a reflection upon the notion of “superiority of a social state” by highlighting the inconsistency of the set {T_N_, ◊C_N_, T_N_ → (C_N_ □ → W_N_), C_N_ □ → $\neg$W_N_}. Thereby, we roughly follow the main ambition Neurath indicates in the original German title of his monograph (1935/1987): how and with which conceptual framework to look at the economy.

The elements of the inconsistent set are:

T_N_: Just like T_U_, the theory T_N_ includes the political and economic background theory Neurath perceives to be dominant. In particular, T_N_ provides an operational definition of the orthodox and dominant notion of superiority, taken to be superiority in terms of monetary calculation (S_M_). Moreover, according to the standard theory T_N_, human well-being and systems of social organization are most fruitfully and adequately evaluated by monetary calculation (S_M_) at the conceptual basis of economics. For relevant cases, well-being in terms of monetary calculation ranks social organizations identically with a more intuitive, pre-scientific notion of superior well-being S_I_. Neurath (1925/2004) reads the standard theory T_N_ to imply extensional interchangeability between superior well-being in terms of monetary calculation S_M_ and superior well-being as adopted in everyday language S_I_.^31^

◊C_N_: As in example U, we have a counterfactual, yet possible, scenario. The properties of this utopian scenario C_N_ and its predicted progression are explored scientifically with a particular focus on the phenomena directly relevant for the notion of superiority S_M_. The outcome O of the utopian scenario is compared to the actual state of affairs A. Let us suppose we confirm the standard theory to the effect that the actual state of affairs A is superior to the utopian outcome O according to the default criterion of monetary calculation S_M_.

T_N_ → (C_N_ □ → W_N_): Let W_N_ be the sentence proclaiming that human well-being is worse in comparison to the actual state of affairs A. Then we can infer: *If T*_*N,*_* then (if C*_*N*_* were implemented, human well-being would deteriorate (relatively)).* Note that the sentence in italics holds because firstly A is S_M_-superior to the utopian outcome O and secondly the background theory T_N_ states that monetary calculation S_M_ is the most fruitful and adequate concept for evaluating human well-being in different social states.

C_N_ □ → $\neg$W_N_: Finally, we mentally explore the utopian scenario described by C_N_. Unlike before, we do not focus solely on phenomena directly relevant to S_M_ but broaden the scope of the investigation. This mental exploration might involve some pre-scientific, everyday language concepts and ideas about well-being. Suppose we discover that the utopian outcome O enhances human well-being as compared to the actual state of affairs A. Due to the proclaimed interchangeability of S_M_ with the more intuitive notion of superiority S_I_, closer to everyday language, this yields the inconsistency of {T_N_, ◊C_N_, T_N_ → (C_N_ □ → W_N_), C_N_ □ → $\neg$W_N_}. The crux of the meta-argument for inconsistency could be explicated like this:
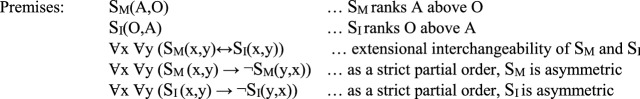
The formulation in first-order predicate logic on the left-hand side allows for a straightforward derivation of the desired conclusion:
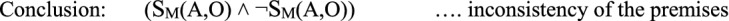


Among the many possibilities of overcoming the inconsistency discussed in example D above, we are most interested in Neurath’s intended conclusion. He would prompt to take the discovery of C_N_ □ → $\neg$W_N_ seriously and to elaborate on its pre-scientific components. Several alternative notions of superiority (S_K1_, S_K2_, S_K3_, …) should be defined by building upon the pre-scientific, everyday-language concepts and ideas used in the thought experiment. S_K1_, S_K2_, S_K3_, … allow for a more thorough investigation of the utopian scenario and a more informative comparison with the actual state of affairs. The precept underlying Neurath’s criticism of the fixation on monetary calculation has been described thus:

Neurath’s methodological axiom was: construct the subject matter you are dealing with in economics—“wealth”, “quality of life”—as an ensemble of heterogeneous elements; do not presume that its heterogeneity might on a deeper level be reduced to one single element (Nemeth 2013, 345–346).

The irreducibility of at least some elements of S_K1_, S_K2_, S_K3_, etc. indicates one last point we want to accentuate: the deliberative aspect of Neurath’s utopianism. Given a multitude of notions of superiority of social states S_K1_, S_K2_, S_K3_, etc., these criteria will rank two social states O and A unanimously only in very rare exceptional cases. In most practically relevant instances, different notions of superiority will point in different directions. For example, O will be superior to A in some respects, say longevity (S_K1_) and leisure hours (S_K4_), while A will be superior to O in other respects, say child mortality (S_K2_) and variety of cultural offers (S_K3_). According to Neurath, a deliberative and democratic process ought to bring about the decision which social state to implement. There will always be a subjective volitional act involved in the weighting of notions of superiority by an individual.^32^ It is only the fixation on one single notion of superiority which triggers the technocratic illusion that science allows a society to dispense with deliberations and decisions.

Neurath’s utopianism is less technocratic than it is sometimes conceived as. He bids the social scientist and the utopianist to contrive various ways of comparison, scientifically apply them to a wide range of different utopias, and present the results. The ideal Neurathian utopianist is not an expert with the authority for deciding on a most “efficient” course of action, but rather an expert for expanding experience to new phenomena and for enlightening the public with adequately presented knowledge for well-informed deliberation. New notions of comparison, like calculation in kind, are conceptual tools for such deliberations (Uebel 2008; see also ﻿Linsbichler 2021; 2022b).

We argued and illustrated that utopias play a crucial role in many thought experiments and that thought experiments involving Neurath’s utopias can—contrary to first doubts—be read as providing arguments. Rest assured, Neurath is no Platonist but a good empiricist.

## Outlook: The Pseudorational Myth of Policies Without Alternatives

What role can thought experiments play in the discovery and justification of new knowledge? This question is particularly pressing for thought experiments in Otto Neurath’s scientific utopianism. In line with Neurath’s logical empiricist stance, we offered a three-fold answer within the boundaries of the empiricist argument view of thought experiments.^33^ Arguments, including arguments which reconstruct the logical structure of a thought experiment, can certainly reveal hitherto unforeseen consequences contained in the premises (and the rules of inference). However, the potential of thought experiments reaches much further. First, regular exercise of imagination and analysis of counterfactual scenarios increase creativity, instil a willingness to question the inevitability of the status quo, and trigger the design and contemplation of new alternative scenarios. Second, the picturesque and vivid mode of presentation of a thought experiment can reinforce its impact and sometimes even induce the construction of new enhanced arguments. Third, utopian thought experiments can challenge the conceptual basis of a scientific discipline. The prompted invention of a new conceptual basis grants access to new phenomena and opens the way to new research questions, genuine discoveries, new knowledge, and in the case of “well-being” perhaps to new policies.

The argument view, especially our reformulation of Häggqvist’s model, accentuates that the logical structure of thought experiments renders them tools which shake our beliefs, our evaluations, and our concepts, including our standards for comparing social systems (see example N in Sect. 4.3). The refined model encourages us to assume and embrace the active role of decision-makers and of architects and explorers of alternative routes, sometimes against common wisdom, embedded cultural practices, or conventions.^34^

Neurath emphasizes that active decisions are unavoidable anyway and that science, for instance, usually cannot uniquely determine which change of a social order is better or more adequate in a given problematic situation, especially if more than one person is involved in the social system (see Neurath 1911/1998; 1912/1973). So, we start with a decision problem and after some thought experiments end up with a decision problem yet again. Nevertheless, scientific utopianism and its thought experiments are not in vain. They can inform and improve deliberations and decisions in various ways: expected consequences of possible policies are investigated and presented in an accessible manner; search for further alternatives and a broader range of available choices is encouraged; and when a thought experiment exposes an inconsistency, all conceived optional reactions can be arrayed in four classes obtainable from our refined version of Häggqvist’s model.

A standard format of four classes of reactions as provided by our refined model could expressly benefit scientific policy advice when it aspires to conform to an ideal of value freedom or when it heeds Neurath’s premonitions against the pseudorational myth of policies without alternatives. Instead of “scientifically” advocating a particular course of action as some commissioned research tends to, proper scientific policy advice in the spirit of Neurath should display the expected consequences of various alternatives. Vivid presentations in the form of thought experiments might be particularly apt for engaging wider audiences to participate in the respective political debates, as long as intuition pumps and other pitfalls are curbed.

In conclusion, by showing how Neurath’s work provides a distinctive perspective to thought experiments which—sometimes sketchily or implicitly—anticipates more meticulous and targeted deliberations by Kuhn, Norton, Gendler, and others, we hope to have shown that scientific utopianism provides tools that can be used in connection with other, more recent tools of philosophical analysis so as to offer new insights into current problems. In other words, this paper attests Neurath’s great significance for current debates in the philosophy of the social sciences, particularly in the philosophy of economics.

## Data Availability

Not applicable.

## Footnotes

1 Note that Norton does not maintain that an appeal to rational reconstruction alone solves all epistemological problems for thought experiments. In Sect. 3.1, we distinguish at least five versions of the claim that thought experiments are “reducible” to arguments.

2 Neurath’s conception of utopia not only diverges significantly from our standard view but also from influential conceptions of his own time. For instance, Neurath (1930/1981) heavily criticizes Mannheim (1929/1936) for neglecting the scientific potential of utopian literature. An exhaustive presentation of scientific utopianism in the context of Neurath’s thought is beyond the aims of this article. Thorough discussions can be found in Uebel 2008; Nemeth & Stadler 1996; Cartwright et al. 1996.

3 According to Neurath, some—but not all—counterfactual scenarios allow for conclusions about reality. Unfortunately, he does not specify any criteria to determine from which models these conclusions can allegedly be drawn or care to elaborate on the detailed workings of such inferences.

4 On the one hand, (ii-b) is weak enough that some authors including Brendel (2018, 291) and Brown (1992) characterize it as running “the risk of amounting to an almost trivially true thesis”. On the other hand, Gendler (2000, 37–38, 47–49) discards the “dispensability thesis” as false, i.e. she negates that any *good scientific* thought experiment can, *without loss of demonstrative force*, be replaced by a non-thought-experimental argument.

5 Häggqvist (2009) rejects the identity thesis (i) for other reasons as well.

6 The question whether one thought experiment corresponds to four argument types or to just one presupposes a well-defined notion of identity for thought experiments, about which there is no consensus (see Bokulich & Frappier 2018).

7 Cf. Neurath (1932/1983, 94): “In unified science we try […] to create a consistent system of protocol sentences and non-protocol sentences (including laws)”.

8 Alternatively, a suitable paraconsistent logic could be adopted or the strive for consistent beliefs could be questioned otherwise. This paper does not consider these hitherto unexplored strategies.

9 Any tool has limited purposes. For other aspects of Neurath’s scientific utopianism, or for the narrative character of utopias (see the Introduction above), Häggqvist’s model might be of no avail.

10 Admittedly, for complex literary works, these reconstructions of utopias, underlying theories, and arguments require countless insertions of intermediate steps and of implicit premises or, alternatively, a very high level of abstraction. Nonetheless, our examples showcase how even sketches of the arguments can reveal the inconsistencies.

11 Variations of the argument might aim at a necessary, possible, or probable instead of a typical outcome.

12 Neurath attacked capitalistic production mostly for being chaotic and unplanned (see Neurath 1925/2004). One might wonder whether Neurath considered these traits as inherently negative or restricted his judgment on the allegedly ensuing effects on social welfare and well-being. Likewise, it could be disputed whether authors on the opposing side of the debate, like Hayek, esteem the unplanned, spontaneous order of the market only for its effects or also partly for its own sake.

13 Cf. Neurath (1931/1983, 89): “A command can never be deduced from a system of statements”.

14 Note that Neurath uses the term “utopia” idiosyncratically without evaluative connotations: “[W]e suggest using the term ‘utopia’ for any kind of invented order, pleasant or unpleasant, plausible or implausible, for maker and reader” (Neurath 1944/1970, 31). Hence, in Neurath’s nomenclature, dystopias are utopias as well.

15 Some utopian novels like Smith’s *The probability broach* work inversely: pleasant surprises challenge initially gloomy suppositions.

16 Throughout the paper, “desirability” is relativized to a group of persons. Thereby, all the sentences in our thought experiments remain *descriptive* and we bypass the many ensuing difficulties of normative sentences in arguments. Indeed, one person’s utopia may be another’s dystopia as H.G. Wells’ *A Modern Utopia* explores (see also Stadler & Neurath 2021). Neurath (1945/2004, 548) also acknowledges the relativity of valuation to persons, for instance when he contemplates people’s expected choices between full employment in a system of “dictatorial planning, based on totalitarian fascism” on the one hand and “painful market society” with “the usual booms and slumps” on the other. The methodological, epistemological, and ontological difficulties of dealing with individual and aggregate valuations, as well as with the logic of normative statements, are beyond the scope of this paper.

17 For atypical readers and atypical protagonists, see strategies 3a and 3b below.

18 If a specific goal or value judgment is revisably presupposed, finding the best decision relative to that goal might become a logical or empirical question. Thus, factual knowledge can and should inform value judgments, particularly for an axiological non-cognitivist like Neurath, “[whose] scientific utopianism remains value-free in that […] sense of refusing to mask value judgments as factual ones. Accordingly, scientific utopianism pursues but instrumental rationality: with certain aims (values) specified, various ways of their implementation are indicated given certain initial conditions” (Uebel 2008, 478). The value-freedom of Neurath’s utopianism notwithstanding, he understands the potential impact of the utopianist’s creative and predictive activities: “[I]f the picture of the future which we design becomes a cause of its own realization, we can justifiably speak of the deliberate shaping of the future” (Neurath 1920/2004, 393–394).

19 For instance, Archard (2017, 24–26) refers to the use of any thought experiments in applied philosophy as prima facie “odd”. A generalized requirement for different standards of impossibility and irrelevance in applied contexts is not justified, provided that the primary concern is the validity of the argument reconstructible from the thought experiment and not the persuasive power of the presentation to a particular audience. We will return to rhetorics in Sect. 4.2.

20 In contrast to Mises, fellow Austrian economist Friedrich Wieser defends an epistemological status for thought experiments closer to Brown’s Platonism (see Tokumaru 2016, 133–153). In any case, Wieser gave prominence to the method of variation in (Austrian) economics. The importance of the method of variation in the work of Mach, the doyen of thought experiments and a forceful influence on Neurath and Austrian economist Hayek, is well known (see e.g. Mach 1906/1917, 183–201).

21 For several imaginary constructions including the evenly rotating economy (ERE), a kind of equilibrium model, Mises argues that in the light of (Austrian) economic theory, they cannot be thought through to their “ultimate logical consequences” (Mises 1949/1998). Mises emphasizes and acknowledges the contradictory nature of ERE, but still stresses its usefulness in arguments (Cowen and Fink 1985). One of the crucial inferences Mises aims to draw is that “the conditions under which the final state of rest is supposed to be established—namely stable conditions—can never be given.” (Hülsmann 2000, 40). In terms of Häggqvist’s model, Mises resolves the discovered inconsistency by maintaining $\neg$◊C_ERE_.

22 Note that this holds not only for deductive, but also for inductive rules of inferences. In a non-formal, but material theory of induction, like Norton’s (2021), the dichotomy between premises and rules of inference may disappear. If the justification provided by a thought experiment is regarded as relative to the premises and the rules of inference, Stuart’s (2016) worries about a tension between the argument view and a material theory of induction are nevertheless mitigated.

23 One could argue that scientific utopianism is merely a form of engineering, a form of intervening in the world, not of learning about it. Such a view ignores that by playing with possibilities, technological research often leads to the acquisition of new, pure knowledge. Neurath clearly embraces this feature of comparative utopistics in his accounts of the “Foundations of Social Science” (Neurath 1944/1970, 30–32) and of his explicitly theoretical (instead of practical) economics (Neurath 1911/1998, 503–504).

24 At the University of Vienna Summer School 2018, *Scientific World Conceptions: Thought Experiments*, both Norton and Brown suggested that an interpreter of thought experiments ultimately must decide between the argument view and concessions to Platonism. Yet, other authors maintain that strictly empiricist alternatives to the argument view exist (Miščević 2018; Nersessian 2018).

25 Emphasizing that utopias are fictional narratives would perhaps be helpful for such studies. But, as stated in the introduction, this is a matter for another paper.

26 Guidelines for particular ways of presentation in Neurath’s ISOTYPE are not solely motivated by psychological arguments about new ways of looking at social issues, but also by methodological considerations like worries about continuous graphs representing discrete phenomena and the demand to depict the quantity of objects by the quantity of signs instead of increasing the size of one sign.

27 What people actually do discover or how they actually revise their beliefs, refers to psychological processes. Judged from Neurath’s strictly logical-empiricist stance, these psychological processes or any intuitions “pumped” by a thought experiment do not play a permissible role in justifying conclusions of thought experiments. Since inflationists and constructivists may disagree, it becomes crucial whether Neurath’s scientific utopianism is explicable and viable in terms of the argument view of thought experiments.

28 Note that the wide conception of ‘logic’ employed by Norton in this context does not strictly require propositional form but merely some type of objects to which rules of inference are applicable (Norton 2004a; Stuart 2016). Other objects than sentences, e.g. models, could be subject to some rules of inference. Most inflationists presumably do not accept any conclusion whatsoever, without caring at all how the (mental) models were manipulated to arrive at the putative conclusion. Yet, as soon as some manipulations of (mental) models are deemed flawed, a reconstruction of the “logical rules for manipulating models” can be attempted and the inflationists’ demur against the argument view is weakened.

29 A response along those lines by champions of the argument view might be regarded as ad hoc by inflationists (e.g., Gendler 2000). Instead, inflationists interpret gaps in reconstructed arguments as evidence against the argument view. Some crucial cognitive or constructive operations in the thought experiment allegedly cannot be faithfully reconstructed in arguments, thus generating the mentioned gaps.

30 Neurath pursues a conscious effort to use scientific utopianism as a means to engender a richer conceptual apparatus in order to lead political economy out of a perceived impasse. In that sense, aspect (III) predates an important feature of the later debates on thought experiments. In his seminal “A Function for Thought Experiments”, Thomas Kuhn (1964/1977) advances that thought experiments are particularly relevant in paradigmatic crises, when scientific communities are looking for creative ways to solve problems without a necessary commitment to the conservation of any well-established standard. This perspective resonates more recently in the works of Tamar Szabó Gendler, who claims that thought experiments promote a reconfiguration of conceptual commitments, directing the attention of thought experimenters to inadequacies in their conceptual schemes and leading them to see exceptional cases through “a different lens” (Gendler 2000, 150–159). Admittedly, it is not prima facie obvious whether Neurath’s proposal is modest or revolutionary. On a modest interpretation, he merely advocates a richer conceptual apparatus but endorses the maintenance of the traditional concepts of monetary calculation for certain niche problems (see e.g. Neurath 1911/1998, 487–489). On a more revolutionary reading, thought experiment N below aims to provoke a reconceptualization after which “some aspects [of the traditional conceptual structure and theory] could only be used after adaptation and augmentation, some had to be eliminated altogether” (Neurath 1917/2004, 312–313). According to Kuhn (1964/1977), a successful revolutionary thought experiment enables “the scientist to use as an integral part of his knowledge what that knowledge had previously made inaccessible to him” (Kuhn 1964/1977, 263) and thereby “learn about the world as well as about his concepts” (261, see also 253). As a strict logical empiricist, Neurath would arguably reject any justificatory role for intuitions “pumped” by the revolutionary thought experiment, but he would, firstly, welcome new conclusions as hypotheses for further logical and empirical investigations, and, secondly, embrace any new arguments as justifications relative to premises and rules of inference. Understanding in further detail how Neurath’s view can be related to Kuhn’s and Gendler’s is a matter for another paper (see Cunha 2022). For now, it suffices as an indication that scientific utopianism can contribute to the contemporary outlook on thought experiments, even in approaches different from the argument view.

31 Actually, the unusual step of explicitly stating that S_M_ is the most fruitful notion of superiority, thereby acknowledging the existence of different notions, is already an approach towards Neurath’s concern—his ever-present criticism of pseudorationalism, the supposition that there is exactly one rational solution to each problem (see also Linsbichler 2021).

32 Prior to that, there is another volitional act involved in the choice of which notions of superiority to define and deploy in different social states. We hold that this volitional act must be guided by interest and pragmatic consideration; albeit, an extreme Platonist might object.

33 The reconstruction thesis of the argument view focuses on the logical structure reconstructible from thought experiments, yet by no means excludes the investigation of psychological, performative, and rhetorical aspects of thought experimental practices.

34 We are grateful to the anonymous reviewers for encouraging us to highlight this point. See also Carrier (2022).
